# Antimicrobial and anti-inflammatory activities of commercial aromatizing fragrances

**DOI:** 10.2144/fsoa-2020-0194

**Published:** 2021-04-07

**Authors:** Hagar Bach, Horacio Bach

**Affiliations:** 1 Faculty of Medicine, Division of Infectious Diseases, University of British Columbia, Vancouver, BC, V6H 3Z6, Canada

**Keywords:** antibacterial, antifungal, essential oils, fragrances, inflammatory response, pathogens

## Abstract

**Aim:**

To explore the bioactivities of commercial fragrances.

**Materials & methods:**

The antimicrobial activity of 25 commercial fragrances was assessed with pathogenic bacteria and fungi in vapor phase. Inflammatory response was evaluated by the measurement of cytokines.

**Results:**

Several fragrances were able to kill the microorganisms. Moreover, preparations of binary mixtures of the most active fragrances showed a synergistic effect. Regarding the inflammatory response, none of the tested fragrances showed a pro-inflammatory response, but two fragrances upregulated the secretion of IL-10 from macrophages.

**Conclusion:**

The antimicrobial activities of fragrances represent a new approach to control airborne pathogens.

## Lay abstract

The demand for indoor aromatizing fragrances has grown over the last few years. Indoors, aromatization is often used to neutralize unpleasant odors produced primarily in enclosed areas with limited ventilation, such as basements and waste storage rooms. In this study, we aim to explore whether commercial fragrances possess bioactivities such as antimicrobial and anti-inflammatory properties. Results showed that several fragrances were able to kill microorganisms tested at different concentrations. The antimicrobial activities of commercial fragrances therefore represent a potential new approach to control pathogens in enclosed and crowded areas, especially for airborne diseases.

The demand for indoor aromatizing fragrances has grown over the last few years. The popularity of these products has increased because of their aromatherapy applications, which claim improvements in people's physical and psychological conditions [1,2]. Moreover, the aromatization of indoor places is often used to neutralize unpleasant odors produced mainly in enclosed areas with limited ventilation, such as basements and waste storage rooms. The generation of these unpleasant odors is aggravated in places with high humidity and temperatures.

According to a recent marketing study, the aromatherapy product global market was estimated at US$6 billion in 2018, with a projection of US$8 billion by the end of 2026 [3].

Most of the products used in aromatherapy are based on essential oils, which are extracted from different parts of plants [4], using steam or microwave-assisted extractions [5,6].

Essential oils are secondary metabolites composed by a complex mixture of volatile organic compounds with around 100 single substances that comprise small organic molecules (<300 Da) belonging mainly to lipophilic terpenoids, phenylpropanoids and short-chain aliphatic derivatives [7].

These compounds undergo chemical changes as the result of different chemical reactions such as oxidation, cyclization and dehydrogenation, causing changes in the start material that can be influenced by the age of the plant, season, soil conditions, weather and location [8].

Essential oils are used in different industrial applications such as food (flavors), perfumes and pharmaceutical preparations, including antiseptics, insect repellents and root canal sealers used in dentistry [9–13]. Moreover, the antimicrobial activity of essential oils has been demonstrated in vapor and liquid phases, supporting their use as food protectants or for therapeutic applications [4,14–18].

The types of ingredients used in the fabrication of commercial fragrances are proprietary formulations composed of a mixture of essential oils, perfumes and other compounds without information about their composition, including essential oil ratios.

The information about human tolerances to these commercial fragrances is scarce and few experimental data have been published. For example, most of the reports focus on high exposure doses or improper use of the fragrances, such as ingestion or skin contact [19]. Furthermore, most of the studies are focused on medical and pharmaceutical applications in the liquid state of the oil, leaving the gaseous phase understudied.

Because essential oils have been reported to have bioactivities, we conducted a study to determine whether commercial fragrances possess antibacterial, antifungal or anti-inflammatory activities.

Here, we report that certain commercial fragrance-containing essential oils used for aromatizing spaces showed antibacterial and antifungal activities against a panel of human pathogens in the gaseous phase. Furthermore, we assessed the immunological response of human macrophages exposed to the fragrances.

## Materials & methods

### Source of fragrances

A total of 25 commercial fragrances were tested in this study. Fourteen of them were generously provided by Doctor Aromas (FL, USA), whereas the rest of the fragrances were purchased from Aroma360 (FL, USA), Aromatech (AZ, USA), Air Esscentials (FL, USA), Scentfinity (NJ, USA), Sage (BC, Canada), Magic Scent (FL, USA) and Aroma Retail (NV, USA) as detailed in Table 1. Doctor Aromas provided a list of the major component of the fragrances for discussion purposes (refer to the ‘Discussion’ section).

**Table 1.** List of the fragrance suppliers used in this study.

Fragrance	Company
1 - Glamour	Doctor Aromas
2 - NFB	Doctor Aromas
3 - SPA	Doctor Aromas
4 - Hope	Doctor Aromas
5 - Caribbean	Doctor Aromas
6 - White Velvet	Doctor Aromas
7 - Oriental Dream	Doctor Aromas
8 - Euphoria	Doctor Aromas
9 - NFC	Doctor Aromas
10 - Success	Doctor Aromas
11 - NFA	Doctor Aromas
12 - SPA (no alcohol)	Doctor Aromas
13 - Wooden Spirit	Doctor Aromas
14 - Antibacterial	Doctor Aromas
15 - Black Velvet	Aroma 360
16 - My Way	Aroma 360
17 - White Tea and Thyme	Aromatech
18 - Sparkling Goji Berry	Air Esscential
19 - Green Tea Lemmongrass	Air Esscential
20 - Fresh Blue	Scentfinity
21 - Gratitude	Sage
22 - Liquid Sunshine	Sage
23 - Heaven	Magic Scent
24 - Secret	Magic Scent
25 - Green Bamboo	Aroma Retail

NFA: New formulation A; NFB: New formulation B; NFC: New formulation C.

### Microbial strains and antimicrobial activities

A panel of human pathogenic bacterial and fungal strains was used in this study. The strains included members of the Gram-positive group *Bacillus subtilis* (ATCC 6633), methicillin-resistant *Staphylococcus aureus* MRSA (ATCC 700698), *Staphylococcus epidermidis* (ATCC 14990), *Listeria monocytogenes* (ATCC BAA-679) and *Staphylococcus aureus* (ATCC 25923). The group of Gram-negative bacteria included *Acinetobacter baumannii* (ATCC BAA-747), *Escherichia coli* (ATCC 25922), *Pseudomonas aeruginosa* (ATCC 14210) and *Salmonella enterica* serovar Typhimurium (ATCC 13311). The panel of pathogenic fungal strains included the filamentous fungi *Aspergillus fumigatus* (ATCC 1022) and *Trichophyton mentagrophytes* (ATCC 18748), whereas *Candida albicans* (ATCC 10231) and *Cryptococcus neoformans* var. *grubii* (kindly provided by Dr. Karen Bartlett, University of British Columbia, BC, Canada) were used as representative of yeast. Bacterial strains were cultured in Mueller-Hinton broth (Becton & Dickinson, NJ, USA) at 37°C overnight at 180 rpm, whereas the fungal strains were cultured in Sabouraud dextrose broth (Becton & Dickinson) at 28°C for a period of 48 or 96 h for yeast and filamentous fungi, respectively. Miller-Hinton and Sabouraud broths were solidified by the addition of 1.5% agar (Becton & Dickinson) and were used for starter cultures and antimicrobial testing.

A starter culture was initiated by growing a single colony in Mueller-Hinton broth for bacteria and Sabouraud broth for yeast. The next day, dilutions of the starters were prepared by diluting the overnight culture in their broths until an optical density of 0.05 (600 nm) was measured following our previous protocols [20]. Harvested spores of the filamentous fungal strains were prepared as previously published [20].

Petri dishes with solidified broths were uniformly spread on their surfaces after soaking a swab with the diluted starter cultures. A sterile rectangular piece of filter paper (1 cm × 1 cm, Whatman #1) was placed on the internal part of the lid and soaked with different volumes of the fragrances. The range of volumes commenced at 100 μl with a succesive decreases by a dilution factor of 1:1 until growth was not observed on the plate. The plates were sealed with parafilm and placed at 37°C for bacterial strains for 18 h or 28°C for fungal strains for 3 days. The volume at which no growth was observed was considered as the minimal inhibitory concentration (MIC) expressed as μl of oil/ml of air. The volume of the atmospheric air entrapped inside the Petri dish was measured after the subtraction of the volume occupied by the solidified broth. Experiments were performed in triplicate.

### Preparation of binary mixtures

Selected fragrances that showed promising results were combined to generate binary mixtures. These mixtures were tested to determine whether a synergistic, additive or antagonist effect would be observed. The ratios used in the binary mixtures are described in Table 2.

**Table 2.** Ratios of binary mixtures.

Fragrances	NFA	NFB	NFC	SPA
A	–	25	75	–
B	–	50	50	–
C	–	75	25	–
D	–	25	–	75
E	–	50	–	50
F	–	75	–	25
G	–	–	25	75
H	–	–	50	50
I	–	–	75	25
J	75	25	–	–
K	50	50	–	–
L	25	75	–	–
M	25	–	75	–
N	50	–	50	–
O	75	–	25	–
P	25	–	–	75
Q	50	–	–	50
R	75	–	–	25

NFA: New formulation A; NFB: New formulation B; NFC: New formulation C.

### Fractional inhibitory concentration

Fractional inhibitory concentration (FIC) values were calculated to determine whether a synergistic or additive effect was exhibited as a result of a combination of fragrances that showed antibacterial or antifungal activities. FIC values were calculated with the equations described below [21].

$$\text{FIC A}=\frac{MIC\;of\;A\;\;in\;the\;presence\;of\;B}{MIC\;of\;A\;individually}$$

$$\text{FIC B}=\frac{MIC\;of\;B\;\;in\;the\;presence\;of\;A}{MIC\;of\;B\;individually}$$

After the FIC values were calculated, the fractional inhibitory concentration index (FIC_Index_) was calculated as follows:

$${\text{FIC}}_{\text{index}}=\text{FIC A}+\text{FIC B}$$

Since FIC_Index_ is calculated based on the MIC of each fragrance in the mixture. A FIC_Index_ <1.0, 1.0 or >1.0 was defined as the synergistic, additive or antagonistic effect of the fragrance combinations, respectively [22].

### Cytotoxicity test

The cytotoxicity activity of the fragrances was measured using the human-derived monocytic cell line THP-1 (ATCC TIB-202). Monocytes were cultured in RPMI (Gibco, Ireland) supplemented with 10% fetal calf serum (Gibco), glutamine and Streptomycin/Penicillin (HyClone, UT, USA) as published [23]. THP-1 cells were dispensed in 96-well plates at a final concentration of 1 × 10^5^ cells/well. Monocytes were differentiated to macrophages after the addition of phorbol-myristate-acetate (30 ng/ml [Sigma-Aldrich, MO, USA]) for 18 h at 37°C in an atmosphere of 5% CO_2_.

The next day, adherent macrophages were treated with a range of fragrance concentrations (0.5–20 μg/ml) diluted in DMSO (Sigma-Aldrich). Cells were treated for 18 h at 37°C in an atmosphere of 5% CO_2_. The cytotoxicity activity was measured using the MTT (3-(4,5-dimethylthiazol-2-yl)-2,5-diphenyltetrazolium bromide) assay as published [24]. Untreated cells and 10% Tween-20 were used as negative and positive controls, respectively. Experiments were performed in triplicate.

### Immunological response

THP-1 cells were used to determine the immunological response after exposure to the fragrances. Macrophages were prepared as mentioned above and treated with the highest concentration of the fragrances that exhibited no cytotoxicity. The next day, the supernatants of the macrophages were collected and used to measure the pro-inflammatory cytokines tutor necrosis factor alpha (TNF-α) and IL-6 and the anti-inflammatory IL-10, using commercial kits (R&D) and following the manufacturer's instructions. Untreated cells and lipopolysaccharide (10 ng/ml from *E. coli*, Sigma-Aldrich) were used as negative and positive controls, respectively. Experiments were performed in triplicate.

### Statistical analysis

A statistical analysis using a t-test (Prism 4, GraphPad Software, Inc.) was performed, and a p-value <0.05 was considered statistically significant.

## Results

### Antimicrobial activities

Three single fragrances (2, 3 and 12) were able to inhibit the growth of six bacterial strains in the range of 1.26–1.68 μg/ml of air, with more activity against Gram-positive (four strains) compared with Gram-negative strains (two strains [Table 3]). None of the fragrances tested showed activity against *P. aeruginosa*. Moreover, nine fragrances showed no antibacterial activity, as indicated in Table 3. Interestingly, *S. epidermidis* and *S.* Typhimurium were susceptible to the fragrances 12 and 14, respectively.

**Table 3.** Antibacterial activity of commercial fragrances expressed as minimal inhibitory concentrations in μg/ml of air.

Fragrance	Bacteria								
	AB	BS	EC	SAL	MRSA	EPI	PA	LIS	SA
1	R	R	R	R	R	R	R	1.68	R
2	1.68	1.26	1.68	R	1.68	R	R	1.68	1.68
3	1.68	1.26	1.68	R	1.26	R	R	1.68	1.68
4	R	R	R	R	R	R	R	1.68	R
5	R	R	R	R	R	R	R	1.68	R
6	R	R	R	R	R	R	R	R	R
7	R	R	R	R	R	R	R	1.68	R
8	R	R	R	R	R	R	R	1.68	R
9	R	1.26	1.68	R	R	R	R	1.68	R
10	R	R	R	R	R	R	R	1.68	R
11	R	1.26	R	R	R	R	R	1.26	R
12	1.68	1.68	1.68	R	1.26	1.68	R	1.68	R
13	R	R	R	R	R	R	R	1.68	R
14	1.68	0.84	1.68	R	1.26	R	R	R	R
15	R	R	R	R	R	R	R	R	R
16	R	R	R	R	R	R	R	R	R
17	R	R	R	R	R	R	R	R	R
18	R	R	R	R	R	R	R	R	R
19	R	1.26	R	R	R	R	R	1.68	R
20	R	1.26	R	R	R	R	R	R	R
21	R	R	R	R	R	R	R	R	R
22	R	R	R	1.68	R	R	R	R	R
23	R	R	R	R	R	R	R	R	R
24	R	R	R	R	R	R	R	R	R
25	R	R	R	R	R	R	R	R	R

AB: *Acinetobacter baumannii*; BS: *Bacillus subtilis*; EC: *Escherichia coli*; EPI: *Staphylococcus epidermidis*; LIS: *Listeria monocytogenes*; MRSA: Methicillin-resistant *Staphylococcus aureus*; PA: *Pseudomonas aeruginosa*; R: Resistant; SA: *Staphylococcus aureus*; SAL: *Salmonella* Thyphimurium.

In the case of the antifungal activities, fragrances showed high activity against the tested fungal strains. For example, all the fragrances showed activity against *T. menthagrophytes* in a range of 0.002–5.0 μg/ml of air (Table 4). The other fungal strains were inhibited by 7 or 8 fragrances at concentrations ranging between 0.84–5.0 μg/ml of air (Table 4).

**Table 4.** Antifungal activity of commercial fragrances expressed as minimal inhibitory concentrations in μg/ml of air.

Fragrance	Fungi			
	AF	CA	CN	TM
1	R	R	R	0.65
2	1.68	1.68	1.68	0.08
3	0.84	0.84	0.84	0.04
4	1.68	R	1.68	0.65
5	R	R	1.68	0.65
6	R	R	R	0.65
7	R	R	R	0.65
8	R	R	R	0.02
9	0.84	0.84	0.84	0.02
10	R	R	R	0.01
11	0.84	0.84	0.84	0.002
12	0.84	0.84	0.84	0.04
13	R	R	R	0.65
14	1.26	1.26	1.26	0.16
15	R	R	R	2.55
16	R	R	R	2.55
17	R	R	R	2.55
18	R	R	R	1.06
19	R	R	R	2.55
20	R	R	R	0.637
21	R	R	R	0.136
22	R	1.68	R	2.55
23	R	R	R	3.82
24	1.27	R	R	5.09
25	5.09	R	R	5.09

AF: *Aspergillus fumigatus*; CA: *Candida albicans*; CN: *Cryptococcus neoformans*; R: Resistant; TM: *Trichophyton mentagrophytes*.

To determine if an interaction occurs between the fragrances that exhibited the inhibition of the highest number of pathogens, binary mixtures were prepared by mixing single fragrances using different ratios of the fragrances 2, 9, 11 and 12 (Table 2).

Results showed that an increase in the antibacterial activity was observed in the binary mixtures. Although the number of bacteria killed by these mixtures was similar to those killed by the single fragrances, a reduction in the MIC values was observed (Table 5). For example, a concentration range between 0.63 and 1.27 μg/ml of air was calculated for the binary mixtures in the Gram-negative group compared with 1.26–1.68 μg/ml of air recorded when the single fragrances were tested. A similar trend was observed in the Gram-positive group. Calculation of the FIC_index_ showed that some of the combinations had synergistic effects, as described in Table 5. The three Gram-negative strains *A. baumannii*, *E. coli* and *P. aeruginosa* were resistant to the binary mixtures. Notwithstanding, some of the mixtures showed antibacterial activities against *S. epidermidis* and *S.* Typhimurium, which were not affected by the single fragrances.

**Table 5.** Antibacterial activity of binary combinations^†^ expressed as minimal inhibitory concentrations in μg/ml of air.

Fragrance	Bacteria								
	AB	BS	EC	SAL	MRSA	EPI	PA	LIS	SA
A	R	R	R	1.27/Ag	R	R	R	R	R
B	R	0.42/S	R	1.27/Ag	0.84/A	R	R	R	R
C	R	0.42/S	R	1.27/Ag	0.84/A	R	R	R	R
D	R	0.84/Ag	R	1.27/Ag	0.84/A	R	R	R	R
E	R	0.84/Ag	R	1.27/Ag	0.84/A	R	R	R	R
F	R	0.84/Ag	R	1.27/Ag	1.26/Ag	R	R	R	R
G	R	0.42/S	R	1.27/Ag	0.42/S	R	R	R	R
H	R	R	R	0.63/Ag	R	R	R	R	R
I	R	0.42/S	R	1.27/Ag	0.84/Ag	R	R	R	R
J	R	0.21/S	R	1.27/Ag	1.26/Ag	1.68/Ag	R	1.26/Ag	1.68/Ag
K	R	0.42/S	R	1.27/Ag	1.26/Ag	1.68/Ag	R	1.26/Ag	1.26/Ag
L	R	0.42/Ag	R	1.27/Ag	1.42/Ag	1.68/Ag	R	0.84/Ag	0.84/Ag
M	R	0.21/S	R	1.27/Ag	1.26/Ag	R	R	1.26/Ag	1.68/Ag
N	R	0.21/S	R	1.27/Ag	1.26/Ag	1.68/Ag	R	1.26/Ag	1.68/Ag
O	R	R	R	1.27/Ag	R	R	R	R	R
P	R	1.26/Ag	R	1.27/Ag	1.26/Ag	R	R	R	1.68/Ag
Q	R	0.21/S	R	1.27/Ag	1.26/Ag	1.68/Ag	R	1.26/Ag	1.68/Ag
R	R	1.26/Ag	R	1.27/Ag	1.26/Ag	1.68/Ag	R	1.26/Ag	1.68/Ag

† Binary mixtures were prepared using different ratios of the fragrances NFA, NFB, NFC and SPA.

A: Additive; AB: *Acinetobacter baumannii*; Ag: Antagonist; BS: *Bacillus subtilis*; EC: *Escherichia coli*; EPI: *Staphylococcus epidermidis*; LIS: *Listeria monocytogenes*; MIC: Minimal inhibitory concentration; MRSA: Methicillin-resistant *Staphylococcus aureus*; NFA: New formulation A; NFB: New formulation B; NFC: New formulation C; PA: *Pseudomonas aeruginosa*; R: Resistant; S: Synergy; SA: *Staphylococcus aureus*; SAL: *Salmonella* Typhimurium.

When the binary mixtures were exposed to fungal strains, all the mixtures except the mixture F for *C. albicans* potentiated the antifungal activities (Table 6).

**Table 6.** Antifungal activity of binary combinations^†^ expressed as minimal inhibitory concentrations in μg/ml of air.

Fragrance	Fungi			
	AF	CA	CN	TM
A	0.21/S	0.84/Ag	0.21/S	0.081/S
B	0.21/S	0.84/Ag	0.42/S	0.081/S
C	0.21/S	0.84/Ag	0.84/Ag	0.16/S
D	0.42/S	0.84/Ag	0.84/Ag	0.081/Ag
E	0.26/S	0.84/Ag	0.84/Ag	0.84/Ag
F	0.11/S	R	0.84/Ag	0.11/Ag
G	0.21/S	0.84/Ag	0.21/S	0.08/Ag
H	0.11/S	0.84/Ag	0.21/S	0.08/Ag
I	0.21/S	0.84/Ag	0.21/S	0.081/Ag
J	0.065/S	0.42/S	0.11/A	0.005/Ag
K	0.32/Ag	0.42/S	0.42/S	0.04/Ag
L	0.42/S	0.84/Ag	0.84/Ag	0.005/Ag
M	0.16/S	0.21/S	0.21/S	0.0025/Ag
N	0.21/S	0.21/S	0.21/S	0.005/Ag
O	0.21/S	0.42/A	0.42/A	0.08/Ag
P	0.42/A	0.42/A	0.42/A	0.04/Ag
Q	0.21/S	0.42/A	0.42/A	0.005/Ag
R	0.21/S	0.21/S	0.21/S	0.005/Ag

† Binary mixtures were prepared using different ratios of the fragrances NFA, NFB, NFC and SPA.

A: Additive; AF: *Aspergillus fumigatus*; Ag: Antagonist; CA: *Candida albicans*; CN: *Cryptococcus neoformans*; NFA: New formulation A; NFB: New formulation B; NFC: New formulation C; R: Resistant; S: Synergy; TM: *Trichophyton mentagrophytes*.

Overall, binary mixtures showed a higher synergistic effect compared with the single fragrances.

### Cytotoxic activity

It is reasonable to think that the presence of oils in the culturing medium (partitioned with dimethyl sulfoxide) would produce the solubilization of membrane macrophages. As a consequence, high cytotoxicity would be measured. Nonetheless, we performed this test to determine the thresholds of the fragrances that showed no cytotoxicity to evaluate the immunological response of the macrophages.

Exposure of the single fragrances to macrophages showed broad variability of the cytotoxicity. For example, most of the single fragrances showed cytotoxic effects when concentrations ranged from 25 to 100 μg/ml (data not shown) were added to the macrophages. The fragrances 2 and 4 showed no cytotoxicity at concentrations <200 μg/ml.

### Immunological response

**Figure 1.**
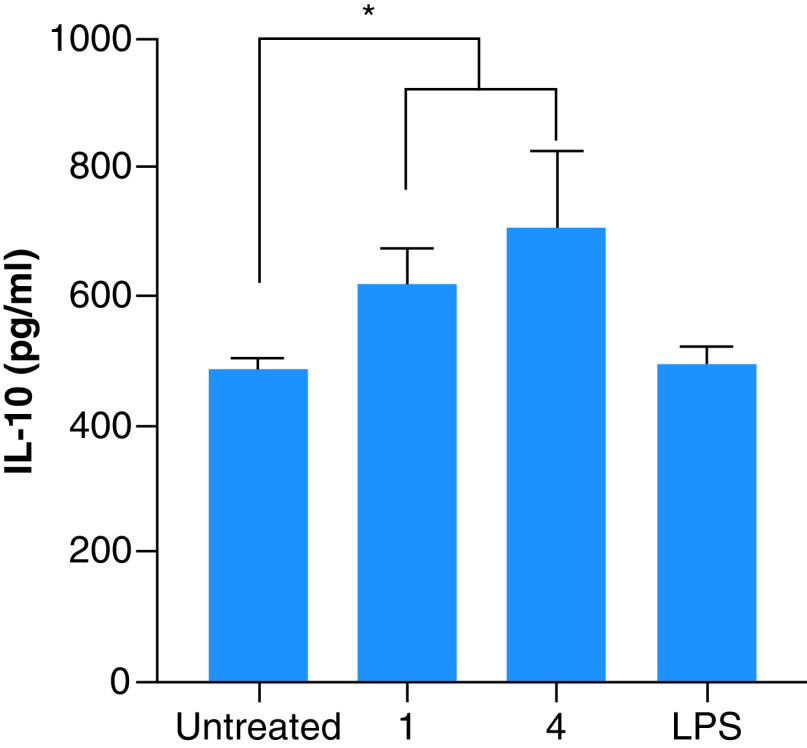
Secretion of IL-10 from macrophages exposed to selected fragrances. Fragrances were exposed to macrophages at concentrations below the cytotoxic level. Only fragrances that showed an increase in the secretion of IL-10 are presented. 1, Glamour; 4, Hope. Untreated macrophages were used as negative controls, whereas lipopolysaccharide was used as a positive control. Experiments were performed according to the Materials & methods. Shown in the mean ± SD of three independent experiments. *p-value <0.05. LPS: Lipopolysaccharide.

The concentrations of the fragrances used to determine the immune response were based on the cytotoxic effects. For example, most of the fragrances were toxic at concentrations >25 μg/ml. Thus, to trigger an immunological response, fragrances concentrations of 20 μg/ml were supplemented to the macrophages. The inflammatory cytokines IL-6 and TNF-α and the anti-inflammatory activity of IL-10 were measured in the supernatant of the cultures. There was no significant increase of the pro-inflammatory cytokines (data not shown), suggesting that the fragrances are not able to elicit an inflammatory response at the concentrations measured. Furthermore, fragrances 1 and 4 showed a significant increase in the levels of IL-10 (Figure 1), suggesting that these fragrances have potential anti-inflammatory properties.

## Discussion

In this study, we aimed to determine whether commercial fragrances used in aromatherapy possess antimicrobial or anti-inflammatory activities.

Commercial fragrances are proprietary mixtures with no disclosed information about their ingredients. Thus, the results of our study cannot be compared with the available literature because of this lack of this knowledge. In the same line, no mechanism of action can be postulated, but knowing that essential oils are part of the fragrances, we can infer a potential mechanism.

Essential oils are mixtures of volatile hydrophobic compounds [25]. Because of their nature, the components do not have a known specific cellular target(s). As typical lipophiles, the essential oil components can permeabilize through the cell wall of the microorganisms, disrupting and damaging the structure of different layers of polysaccharides, fatty acids, membranes and phospholipids [26,27].

In bacteria, the permeabilization of the lipophiles may destabilize membranes with detrimental effects associated with loss of ions, leading to a reduction of the membrane potential, the collapse of the proton pump, the depletion of the ATP pool, with cytoplasm leakage and cell lysis. For example, phenolic compounds are abundant in essential oils and have been shown to denature proteins in Gram-positive bacteria [28]. Other mechanisms of action include general observations rather than specific pathways such as an increase in the membrane permeability [29–31], enzyme inhibition [32,33], leakage [34], essential metal leakage [35], proton exchange disruption [36] and a reduction of the intracellular pH [37–39].

In the present study, we found that the fragrances are less efficient to kill Gram-negative bacteria. This difference from Gram-positive bacteria can be attributed to the cell composition that differentiates both groups. Specifically, Gram-negative bacteria possess a double cell wall separated by the periplasm. Thus, the periplasm might play a buffer zone, slowing down the permeabilization of the compounds responsible for the antibacterial activity compared with the single-cell wall of Gram-positive bacteria.

In the case of fungal cells, the lipophiles, upon permeabilization, can interact with different types of intracellular membranes [40,41]. For example, impairing the mitochondrial membranes by depolarization will render the cell with depleted ATP, leading to cell death resulting from apoptosis and necrosis. Another way to exert cell death is by altering membrane potential will affect the influx/efflux of essential ions, causing a reduction in pH gradient, with detrimental effect to the proton pump and the ATP storage [42,43].

In this study, we report that the fragrances efficiently controlled the filamentous fungus *A. fumigatus*. The genera *Aspergillus* comprises many strains defined as molds that can grow in many types of surfaces. The presence of molds represents a health concern because of their ability to cause allergies and even cancer development as a result of the production of aflatoxins [44,45]. Thus, the antifungal activities of the fragrances can be exploited for the control of molds in many places, including basements; humid rooms with or without proper ventilation and can be dispensed through air-conditioning systems for homogeneous distributions of the essential oil components.

Analyses of the compounds in the fragrances with antimicrobial activity were investigated, focusing on the fragrances that showed inhibition to the highest number of bacterial strains. For example, the fragrances 2, 3, 12 and 14 (Table 3) inhibited the growth of six pathogenic strains. These fragrances contain menthol and citral, which have been broadly reported as antibacterial compounds [46–49]. In addition, the same fragrances above mentioned showed antifungal activities against the four fungal pathogenic strains tested in this study (Table 4), confirming their potential role as the antifungal component of the fragrances as demonstrated by other research groups [50–54].

The fragrances 9 and 11 exhibited antifungal activity as well (Table 4). In fragrance 9, the identified compound was menthyl acetate. There was no antifungal activity information in the literature to relate its direct role as an antifungal compound. However, in fragrance 11, the antifungal activity can be attributed to the presence of geranial and neral, which have been reported as antifungal compounds [55–58].

Regarding the inflammatory response, no fragrance showed a significant increase in the level of the pro-inflammatory cytokines IL-6 and TNF-α (data not shown). However, the fragrances 1 and 4 significantly increased the secretion of the anti-inflammatory cytokine IL-10 by an unknown mechanism based on the information of the fragrances (provided by the manufacturer), both 1 and 4 contain D-limonene and linalool. In line with our study, previous studies reported that D-limonene [59,60] and linalool [61,62] have showed anti-inflammatory activities by increasing the levels of IL-10. This observation is of high importance because secretion of IL-10 in the lungs is desired for people with asthma [63]. More studies are necessary to validate this observation in animal models.

## Conclusion

In summary, to the best of our knowledge, this is the first study reporting antimicrobial and anti-inflammatory activities of commercial fragrances. The antimicrobial activities of commercial fragrances represent a new approach to control pathogens in enclosed and crowded areas, especially for airborne-related diseases. Also, the fact that two fragrances showed a significant upregulation in the secretion of IL-10, more studies *in vivo* are necessary to confirm this finding as a potential adjuvant for asthma treatment.

## Future perspective

In this study, we present the findings that commercial fragrances possess antimicrobial activities. The fact that fragrances consumption is becoming more popular is possibly suggestive of their dual activity: neutralization of unpleasant odors and to control microorganisms. Future efforts should be focused on the proper distribution of these fragrances to sanitize potential disseminators of microbial pathogens such as air conditioning units in shopping centers, hospitals, or any place where a conglomeration of people might be affected by these microbes.

**Summary points**

- The demand and consumption of fragrances to aromatize ambients has steadily increased over the last years.
- We demonstrate that commercial fragrances possess antimicrobial activities against pathogens.
- Commercial fragrances also enhanced the secretion of the anti-inflammatory cytokine IL-10.
- The use of fragrances in air conditioning distribution could represent a new method of sanitation.
